# Supplementary material for: Assessment of the Methods Used to Develop Vitamin D and Calcium Recommendations—A Systematic Review of Bone Health Guidelines
Source: Nutrients. 2021 Jul 15;13(7):2423. doi: 10.3390/nu13072423 (PMC8308557; doi:10.3390/nu13072423)
Supplement: Supplementary file 1 [file nutrients-13-02423-s001.zip › nutrients-1266131-supplementary.pdf]

Assessment of the methods used to develop vitamin D and calcium recommendations – a systematic review of bone health guidelines

Zhaoli Dai et al.

Supplementary Material

Supplementary Table S1. Description of WHO methodological criteria for guideline development

Methods domains	Processes and methods	Description
I. Guideline development group (GDG, including members of steering group, research team and individuals involved formulating the final recommendations)		
Were each of the following considered in the formation of the guideline development group?	1. Discipline representation	Information about the composition, discipline, and relevant expertise of the guideline development group should be provided.
	2. Diversity representation	Information about gender, diversity, across the life-course, subject to different gender norms, and belonging to different income and education groups of the guideline development group.
	3. Stakeholder input	Stakeholders such as nongovernmental organizations, advocacy groups, funders, target audiences, and service-users may be invited to ensure transparency of the processes and facilitate implementation.
Were each of the following steps addressed regarding conflicts of interest?	4. Disclosure of conflicts of interest obtained (extract verbatim text of COI for each member)	Is there an explicit statement that all group members have declared whether they have any competing interests?
	5. Conflicts of interest managed	Members declaration of interests must be reported to the steering group. Potential candidates for membership who have major conflicts of interest, be they financial or nonfinancial, cannot be appointed to the G.D.G. Minor conflicts of interest can be managed at the

		individual level (e.g. by restricting participation in parts of the GDG meeting) or at the group level.
	6. Disclosure of funders of the guideline obtained and disclose funder's role in influencing the guideline development process and recommendations	Is there an explicit statement of funder of the guideline and the role of funders in the final guideline recommendations?
Were methods for each of the following addressed in the guideline?	7. Formulation of key questions for the evidence review in PICO, PICOT, or PEO format (also extract the key questions in such format)	Key questions are framed in a way that enables a systematic search of the literature and delineates inclusion and exclusion criteria for the body of evidence to formulate the research questions for the recommendations in such format.
	8. Choosing (finalizing) priority outcomes for systematic review	List high-priority key questions and the outcomes to formulate recommendations.
	9. Systematic methods to search for evidence	Details of the strategy used to search for evidence should be provided including search terms used, sources consulted, and dates of the literature covered. Sources may include electronic databases, hand searching journals, reviewing conference proceedings, and other guidelines.
	10. Evidence retrieval (screening and selection of eligible studies)	Process of data from eligible studies are extracted and search strategy and results should be carefully documented.
	11. Evidence quality assessment	Each study included in a systematic review should be assessed for risk of bias (e.g. use the Cochrane risk of bias tool, Quality assessment tools project report, etc.)
	12. Evidence synthesis	The findings of the systematic review may be synthesized in a narrative manner or quantitatively in a meta-analysis. The review should describe how data were handled and why a given approach to synthesis was taken for each outcome.
If evidence was explicitly linked to recommendation, what type of evidence was reported?	13. Are recommendations explicitly linked to evidence?	An explicit link between the recommendations and the evidence on which they are based should be included in the guideline. The guideline user should be able to identify the components of the body of evidence relevant to each recommendation.

Was each of the following items considered when developing the recommendation?	14. Was a consensus process clearly described for developing recommendations	A description of the methods used to formulate the recommendations and how final decisions were arrived at should be provided. For example, methods may include a voting system, informal consensus, and formal consensus techniques. Areas of disagreement and methods of resolving them should be specified.
	15. Was a method employed to determine strength and/or certainty of the recommendation?	Is there a method provided to influence the direction and strength of a recommendation (e.g. use GRADE framework and others)
	16. Priority of the problem: Is the problem a burden of disease?	The problem's priority is determined by its importance and frequency (i.e. burden of disease, disease prevalence or baseline risk). The greater the importance of the problem, the greater the likelihood of a strong recommendation.
	17. Quality of the evidence: Is higher quality of the body of evidence included to support the recommendation?	Is there a method provided to grade the quality of body of evidence to assess the strength of the recommendation (e.g. GRADE and others)
	18. Certainty of evidence: Does the recommendation include consistent body of evidence (e.g. confidence in effect estimates)?	The quality of the evidence – the degree of confidence in the estimates of effect. This is a key factor in determining the strength of a recommendation.
	19. Benefits and harms: Are evaluations performed on the net benefit or net harm associated with an intervention or exposure?	The balance between an intervention's or exposure's benefits and harms. Did the guideline development group consider the magnitude of the effects and the relative importance of the outcomes, including any disadvantages or inconveniences associated with the intervention.
	20. Balance: Does the balance between desirable and undesirable effects support the recommendation?	Does the balance between desirable and undesirable effects favour the intervention or the comparison?

	21. Outcome importance: Is there important uncertainty about or variability in how much people value the main outcome?	Is there important uncertainty about or variability in how much people value the main outcomes, including adverse effects and burden of the test and downstream outcomes of clinical management that is guided by the test results?
	22. Equity: Does the evidence used reduce inequalities, improve equity or contribute to the realization of one of several human rights defined under the international legal framework?	What would be the impact on health equity?
	23. Acceptability: Is the option acceptable to key stakeholders?	A strategy to address concerns about acceptability during implementation will be included in the guideline with the recommendations. Acceptability is affected by several factors, such as who benefits from an intervention and who is harmed by it; who pays for it or saves money on account of it; and when the benefits, harms and costs occur.
	24. Feasibility: Is the option feasible to implement?	Feasibility is influenced by the resources available, programmatic considerations, the existing and the necessary infrastructure and training, etc.
	25. Was the guideline/recommendation reviewed by an external review group?	Is there an explicit statement about the peer review of the draft final guideline? The external review group is composed of persons interested in the subject of the guideline as well as individuals who will be affected by the recommendations.

Supplemental Table S2. Included guidelines in the systematic review

Country of origin	Published year	Title of guideline
Australia	2010	Clinical guideline for the prevention and treatment of osteoporosis in postmenopausal women and older men (1)
Australia and New Zealand	2009	Calcium and bone health: position statement for the Australian and New Zealand Bone and Mineral Society, Osteoporosis Australia and the Endocrine Society of Australia (2)
Belgium	2011	Non-pharmacological management of osteoporosis: A consensus of the Belgian Bone Club (3)
Brazil	2017	Recommendations of the Brazilian Society of Endocrinology and Metabology (SBEM) for the diagnosis and treatment of hypovitaminosis D (4)
Canada	2010	Vitamin D in adult health and disease: a review and guideline statement from Osteoporosis Canada (5)
Canada	2010	2010 clinical practice guidelines for the diagnosis and management of osteoporosis in Canada: summary (6)
Canada	2014	Society of Obstetricians and Gynaecologists of Canada for Osteoporosis in Menopause(7)
Central Europe	2013	Practical guidelines for the supplementation of vitamin D and the treatment of deficits in Central Europe (8)
Europe	2014	Management of osteoporosis of the oldest old.(9)
Europe	2014	The role of dietary protein and vitamin D in maintaining musculoskeletal health in postmenopausal women: A consensus statement from the European Society for Clinical and Economic Aspects of Osteoporosis and Osteoarthritis (ESCEO) (10)
Europe	2013	Vitamin D supplementation in elderly or postmenopausal women: a 2013 update of the 2008 recommendations from the European Society for Clinical and Economic Aspects of Osteoporosis and Osteoarthritis (ESCEO) (11)
Europe	2019	Current vitamin D status in European and Middle East countries and strategies to prevent vitamin D deficiency: a position statement of the European Calcified Tissue Society (12)
Greece	2012	2011 Guidelines for the Diagnosis and Treatment of Osteoporosis in Greece (13)
Hong Kong	2013	2013 OSHK Guideline for the clinical management of postmenopausal osteoporosis in Hong Kong (14)
Ibero-America	2009	"IberoAmerican Consensus on Osteoporosis Sibomm 2009" Osteoporosis: Prevention, Diagnosis, and Treatment (15)
India	2013	Clinical practice guidelines on menopause: *An executive summary and recommendations (16)
International	2010	IOF position statement: vitamin D recommendations for older adults (17)
International	2019	Executive summary of European guidance for the diagnosis and management of osteoporosis in postmenopausal women (18)
Italy	2016	Guidelines for the diagnosis, prevention and management of osteoporosis (19)

Japan	2012	Japanese 2011 guidelines for prevention and treatment of osteoporosis-executive summary (20)
Jordan	2016	The Practical Guide for Management of Osteoporosis (21)
Malaysia	2013	The Malaysian Clinical Guidance on the Management of Postmenopausal Osteoporosis, 2012 (22)
Pan Arab	2017	Pan Arab Osteoporosis Society Guidelines for Osteoporosis Management (23)
Philippines	2011	Consensus statements on osteoporosis diagnosis, prevention, and management in the Philippines (24)
Poland	2018	Vitamin D Supplementation Guidelines for General Population and Groups at Risk of Vitamin D Deficiency in Poland (25)
Poland	2017	Guidelines for the diagnosis and management of osteoporosis in Poland (26)
Saudi Arabia	2011	Updated Recommendations for the Diagnosis and Management of Osteoporosis: A Local Perspective (27)
Singapore	2012	A Review of the 2008 Singapore Ministry of Health Clinical Practice Guidelines on Osteoporosis and an Update (28)
South Africa	2010	NOFSA statement on the appropriate management of osteoporosis in the elderly (29)
Spain	2017	Recommended vitamin D levels in the general population (Spain) (30)
Taiwan	2013	Taiwanese Guidelines for the Prevention and Treatment of Osteoporosis (31)
Taiwan	2014	Consensus of Official Position of IOF/ISCD FRAX Initiatives in Asia-Pacific Region (32)
Thailand	2016	Thai Osteoporosis Foundation (TOPF) position statements on management of osteoporosis (33)
UK.	2014	Vitamin D: supplement use in specific population groups (34)
UK.	2016	Sunlight exposure: risks and benefits (35)
UK.	2017	UK clinical guideline for the prevention and treatment of osteoporosis (36)
UK.	2018	Osteoporosis Clinical guideline for prevention and treatment (37)
UK.	2013	Diagnosis and management of osteoporosis in postmenopausal women and older men in the UK: National Osteoporosis Guideline Group (NOGG) update 2013 (38)
US.	2018	Vitamin D, Calcium, or Combined Supplementation for the Primary Prevention of Fractures in Community-Dwelling Adults US Preventive Services Task Force Recommendation Statement (39)
US.	2012	Osteoporosis in Men: An Endocrine Society Clinical Practice Guideline (40)
US.	2014	Recommendations Abstracted from the American Geriatrics Society Consensus Statement on Vitamin D for Prevention of Falls and Their Consequences (41)
US.	2010	The North American Menopause Society**.** Management of osteoporosis in postmenopausal women (42)
US.	2014	Clinician's Guide to Prevention and Treatment of Osteoporosis (43)

US.	2016	American Association of Clinical Endocrinologists and American College of Endocrinology Clinical Practice Guidelines for the Diagnosis and Treatment of Postmenopausal Osteoporosis – 2016 (44)
US.	2009	Diagnosis and Treatment of Osteoporosis (45)
US.	2013	ACOG Releases Practice Bulletin on Osteoporosis (46)
Vietnam	2015	Calcium and Vitamin D Deficiency in Vietnamese: Recommendations for an intervention strategy (47)

Supplemental Table S3: Exclusion of full-text records

**Full-text articles excluded (n=70)**	**Title of article**	**Reason to exclude**	**Year published**	**Reference**
**Out of scope (n=13)**	Following NICE guidance to take positive steps to prevent falls	Other conditions; educational materials to nurses	2013	(48)
	Taking control of osteoporosis to cut down on risk of fracture	Educational materials to nurses	2011	(49)
	Health disparities in endocrine disorders: biological, clinical, and nonclinical factors--an Endocrine Society scientific statement	Other conditions	2012	(50)
	Vitamin D: Implications of the Institute of Medicine Report for Clinical Practice	Educational materials to nurses	2012	(51)
	Following MyPyramid the Plant-Based Way: Effects on Food Patterns and Nutrients	Other topics, on food patterns	2012	(52)
	Lack of Evidence Linking Calcium With or Without Vitamin D Supplementation to Cardiovascular Disease in Generally Healthy Adults: A Clinical Guideline From the National Osteoporosis Foundation and the American Society for Preventive Cardiology	Other conditions	2016	(53)
	Treatment of Osteoporosis in Australian Residential Aged Care Facilities: Update on Consensus Recommendations for Fracture Prevention	Special group of population	2016	(54)
	Osteoporosis in Frail Patients: A Consensus Paper of the Belgian Bone Club	Special group of population	2017	(55)
	Reducing falls risk and medication reviews	Other conditions and medications	2009	(56)

	Extraskeletal benefits and risks of calcium, vitamin D and anti-osteoporosis medications	Other conditions and medications	2012	(57)
	Diagnosis and Treatment of Osteoporosis in Older Adults	Drug review	2013	(58)
	Interventions to prevent falls in community-dwelling older adults: Recommendation statement	Other conditions	2018	(59)
	Prescribing for osteoporosis	Medications	2010	(60)
**Duplicates (6)**	Screening for osteoporosis: U.S. preventive services task force recommendation statement	Older version of the USPSTF recommendations (used 2018 version in analysis)	2011	(61)
	Guidelines for the diagnosis and management of osteoporosis in postmenopausal women and men from the age of 50 years in the UK	Older version of the UK National Osteoporosis Guideline Group (used 2013 version in analysis)	2009	(62)
	Three new recommendations from the US Preventive Services Task Force	Older version of the USPSTF recommendations (used 2018 version in analysis)	2013	(63)
	Polish guidelines for the diagnosis and management of osteoporosis: a review of 2013 update	Older version of "Guidelines for the diagnosis and management of osteoporosis in Poland" (used 2017 version in analysis)	2014	(64)
	Vitamin D in adult health and disease: a review and guideline statement from Osteoporosis Canada (duplicates)	Duplicate of 2010 clinical practice guidelines for the diagnosis and management of osteoporosis in Canada	2010	(65)
	Vitamin D and calcium supplementation to prevent fractures in adults: U.S. Preventive Services Task Force recommendation statement	Older version of "Vitamin D, Calcium, or Combined Supplementation for the Primary Prevention of Fractures in Community-Dwelling Adults US Preventive Services Task Force	2013	(66)

		Recommendation Statement" (used 2018 version in analysis)		
**Primary study (n=16)**	Efficacy of a dose range of simulated sunlight exposures in raising vitamin D status in South Asian adults: implications for targeted guidance on sun exposure	An observational study	2013	(67)
	Vitamin D and Calcium Supplementation to Prevent Fractures in Adults	A case study	2014	(68)
	Variation in Vitamin D supplementation among adults in a multi-race/ethnic health plan population, 2008	A health survey	2012	(69)
	Are commonly recommended dosages for vitamin D supplementation too low? Vitamin D status and effects of supplementation on serum 25-hydroxyvitamin D levels-an observational study during clinical practice conditions	An observational study	2011	(70)
	The incidence of vitamin D deficiency amongst patients with a femoral neck fracture: are current bone protection guidelines sufficient?	An observational study	2013	(71)
	Hip fracture prevention strategies in long-term care: a survey of Canadian physicians' opinions	A survey	2010	(72)
	The potential impact of new National Osteoporosis Foundation guidance on treatment patterns	An observational study	2010	(73)
	A baseline audit to evaluate the quality of nutritional provision delivered to fractured neck of femur inpatients (case study)	A case study	2011	(74)
	Preventing fractures in postmenopausal women: How to assess risk	A case study	2011	(75)

	Sun exposure guidelines and serum vitamin D status in Denmark: The status D study	An observational study	2016	(76)
	Checklist for prevention of new hip fractures	A before and after comparison study	2013	(77)
	Bone mineral density screening and its accordance with Canadian clinical practice guidelines from 2000-2013: an unchanging landscape in Saskatchewan, Canada	An observational study	2015	(78)
	Management of hip fractures in older people in Beijing: a retrospective audit and comparison with evidence-based guidelines and practice in the UK	An observational study	2016	(79)
	Screening, prevention and management of osteoporosis among Canadian adults	An observational study	2018	(80)
	Recommended summer sunlight exposure levels can produce sufficient (> or =20 ng ml (-1)) but not the proposed optimal (> or =32 ng ml (-1)) 25(OH)D levels at UK latitudes	An observational study	2010	(81)
	Vitamin D and musculoskeletal health, cardiovascular disease, autoimmunity and cancer: Recommendations for clinical practice	A panel interview	2010	(82)
**Reviews (n= 10)**	Vitamin D: beneficial for pain, fracture, and falls in long-term care residents?	A review	2011	(83)
	Calcium and bone health--goodbye, calcium supplements?	A review	2015	(84)
	Vitamin D deficiency insufficiency and challenges in developing global vitamin D fortification and supplementation policy in adults	A systematic review	2012	(85)

	Vitamin D in the Middle East and North Africa	A review	2018	(86)
	Calcium and vitamin D supplementation on bone health: Current evidence and recommendations	A review	2014	(87)
	The calcium scare-what would Austin Bradford Hill have thought?	A review	2011	(88)
	Vitamin D supplementation in adults	A review	2010	(89)
	Identifying and managing osteoporosis: an update (algorithm)	A review	2010	(90)
	Prevention and Treatment of Osteoporosis in Postmenopausal Women: a review...Part two	A review	2011	(91)
	Osteoporosis and fracture risk in older people	A review	2014	(92)
**Commentary (n=19)**	Bisphosphonate and vitamin D supplementation for osteoporosis	A commentary	2012	(93)
	The diagnosis and treatment of primary osteoporosis according to current guidelines: impairments precede fractures...CME article about '... most common generalized disease of the skeleton... '	A commentary	2009	(94)
	Here comes the sun: good news for bone health!	A commentary	2011	(95)
	Triennial Growth Symposium--Basis for establishment of 2011 vitamin D guidelines in humans	A commentary	2014	(96)
	Minerals and vitamins in bone health: the potential value of dietary enhancement	A commentary	2009	(97)
	Clinical practice. Vitamin D insufficiency	A commentary/correspondence	2011	(98)

	Vitamin D insufficiency: Definition, diagnosis and management	A commentary	2018	(99)
	Building healthy bones throughout life: an evidence-informed strategy to prevent osteoporosis in Australia	A commentary	2013	(100)
	Vitamin D status and its management for achieving optimal health benefits in the elderly	A commentary	2018	(101)
	Health impacts of vitamin D: are we getting enough?	A commentary	2009	(102)
	Clinical Review: The 2011 report on dietary reference intake for vitamin D: where do we go from here?	A commentary	2011	(103)
	USPSTF: Taking Vitamin D and Calcium Doesn't Prevent Fractures in Older Women	A commentary	2012	(104)
	Managing osteoporosis in postmenopausal women	A commentary	2010	(105)
	Vitamin D and Calcium: Implications for Healthy Aging	A commentary	2011	(106)
	Clinical Q&A	A commentary	2010	(107)
	Vitamin D deficiency	A commentary	2015	(108)
	Osteoporosis management in 2017: still thin and fragmented	A commentary	2017	(109)
	Calcium supplements: do they help or harm?	A commentary	2014	(110)
	The new calcium and vitamin D DRIs: a clinician's guide	A commentary	2011	(111)
No recommendations on vitamin D or calcium (n=3)	Final Declaration of the Asian Metaforum on the Role of Vitamin D and the Management of Osteoporosis	No intake level of vitamin D provided	2009	(112)

	Osteoporosis - National Institute for Health and Care Excellence	No recommendations on vitamin D or calcium	2017	(113)
	Hip fracture - National Institute for Health and Care Excellence	No recommendations on vitamin D or calcium	2012	(114)
Non-English articles (n=2)	The Chinese osteoporosis guideline (2017)	Text published in Chinese	2017	(115)
	Guidelines for the diagnosis, prevention and treatment of osteoporosis, 2012	Text published in Spanish	2013	(116)
No full text available (n=1)	Recommendations for the diagnosis, prevention and treatment of vitamin D insufficiency and deficiency	Only abstract is available	2013	(117)

Supplemental Table S4. Recommendations on vitamin D and calcium in the top 10 bone health guidelines that met at least 16 of the 25 WHO methodological criteria for guideline development

Guideline title	Country/ Region	Year	Vitamin D recommendations	Calcium recommendations
Clinical guideline for the prevention and treatment of osteoporosis in postmenopausal women and older men (1)	Australia	2010	"If vitamin D supplements are required, doses of at least 800 IU/day are usually required."	"Total calcium intake from dietary sources and supplements should exceed 1200 mg/day."
2010 clinical practice guidelines for the diagnosis and management of osteoporosis in Canada: summary (6)	Canada	2010	"For healthy adults at low risk of vitamin D deficiency, routine supplementation with 400-1000 IU (10-25 μg) vitamin D3 daily is recommended [grade D]." "For adults over age 50 at moderate risk of vitamin D deficiency, supplementation with 800-1000 IU (20-25 μg) vita min D3 daily is recommended. To achieve optimal vitamin D status, daily supplementation with more than 1000 IU (25 μg) may be required. To achieve optimal vita - min D status, daily supplementation with more than 1000 IU (25 μg) may be required."	"The total daily intake of elemental calcium (through diet and supplements) for individuals over age 50 should be 1200 mg [grade B]."
Vitamin D in adult health and disease: a review and guideline statement from Osteoporosis Canada (5)	Canada	2010	"In healthy adults at low risk for vitamin D deficiency (i.e., under age 50, without osteoporosis or conditions affecting vitamin D absorption or action), routine vitamin D supplementation (10-25 μg [400-1000 IU] daily) is recommended." "Adults over 50 years of age are at moderate risk for vitamin D deficiency. Supplementation	"Vitamin D3 at daily doses of 20 μg (800 IU), in combination with calcium (1000 mg), reduces the risk of hip and nonvertebral fractures in elderly people living in institutions (level 1 evidence). The evidence for community dwelling individuals is less strong (level 2 evidence)."

			with at least 20-25µg (800-1000 IU) of vitamin D3 daily is recommended."	
Consensus statements on osteoporosis diagnosis, prevention, and management in the Philippines (24)	Philippines	2011	“Reference Nutrient Intake: Male 50-64 y 10 µg/day; 65 and over 15 µg/day; women 19-49y: 15 µg/day; 50y and over: 10-15 µg/day ”. “However, it is not routinely recommended to give vitamin D analogues.”	"It is recommended that postmenopausal women take at least 750-800 mg of calcium daily for minimum of 2 years to prevent bone loss. The magnitude of reduction in fracture risk with calcium supplementation alone remains unclear. It is also recommended that dose of 10-20 µg of vitamin D3 be given with 1000 mg calcium to reduce hip fracture risk of older individuals especially those with limited sun exposure, i.e. housebound.”
NOFSA Guideline for the Diagnosis and Management of Osteoporosis (29)	South Africa	2010	Ensure an adequate intake of calcium (1,200mg per day) and vitamin D (800-1,000 IU per day)”	“Adult women and men 25 - 65 years 1,000 mg/day; Over 65 years 1,200 mg /day”
Vitamin D: supplement use in specific population groups (34)	U.K.	2014	“They recommend that vitamin D supplements are made available for the entire population (4 years old or more) throughout the year. The recommendations should be read in conjunction with The SACN vitamin D and health report 2016: 2-4 µg/d (80-160 IU/d) for ages 1.5-64y; 5 µg/d (200 IU/d) for adults aged ⩾ 65y”	n/a
U.K. clinical guideline for the prevention and	U.K.	2017	"It is recommended that in postmenopausal women and men ⩾50 years who are at increased risk of fracture, a daily dose of	"It is recommended that a daily calcium intake of between 700 and 1200 mg should be advised, if

treatment of osteoporosis (36)			800 IU of cholecalciferol should be advised (Grad A recommendation" "In postmenopausal women and older men (⩾ 50 years) at increased risk of fracture a daily dose of 800 IU cholecalciferol should be advised."	possible achieved through dietary intake (Grade B recommendation)."
Vitamin D, Calcium, or Combined Supplementation for the Primary Prevention of Fractures in Community-Dwelling Adults U.S. Preventive Services Task Force Recommendation Statement (39)	U.S.	2018	"The current recommendation against supplementation with 400 IU or less of vitamin D and 1000 mg or less of calcium for the primary prevention of fractures is primarily based on the finding of no benefit with supplementation at lower doses."	"… and 1000 mg or less of calcium for the primary prevention of fractures is primarily based on the finding of no benefit with supplementation at lower doses."
Osteoporosis in Men: An Endocrine Society Clinical Practice Guideline (40)	U.S.	2012	"Vitamin D (recommended daily intake, 10 to 20 μg)"	"We recommend that men with or at risk for osteoporosis consume 1000-1200 mg calcium daily, ideally from dietary sources, with calcium supplements added if dietary calcium is insufficient."
Recommendations Abstracted from the American Geriatrics Society Consensus Statement on Vitamin D for Prevention of Falls and Their Consequences (41)	U.S.	2014	"Clinicians are strongly advised to recommend vitamin D supplementation of at least 1,000 IU/d with calcium to older adults residing in institutionalized settings to reduce the risk of fracture and falls."	"Calcium: … with 1,000 to 1,200 mg/d most commonly prescribed."

Supplemental references

1. The Royal Australian College of General Practitioners. Clinical guideline for the prevention and treatment of osteoporosis in postmenopausal women and older men. 2010.

2. Sanders KM, Nowson CA, Kotowicz MA, Briffa K, Devine A, Reid IR, Working group A, New Zealand B, Mineral S, Osteoporosis A. Calcium and bone health: position statement for the Australian and New Zealand Bone and Mineral Society, Osteoporosis Australia and the Endocrine Society of Australia. Med J Aust 2009;190(6):316-20.

3. Body JJ, Bergmann P, Boonen S, Boutsen Y, Bruyere O, Devogelaer JP, Goemaere S, Hollevoet N, Kaufman JM, Milisen K, et al. Non-pharmacological management of osteoporosis: A consensus of the Belgian Bone Club. Osteoporosis International 2011;22(11):2769-88. doi: http://dx.doi.org/10.1007/s00198-011-1545-x.

4. Maeda SS, Borba VZ, Camargo MB, Silva DM, Borges JL, Bandeira F, Lazaretti-Castro M. Recommendations of the Brazilian Society of Endocrinology and Metabology (SBEM) for the diagnosis and treatment of hypovitaminosis D. Arq Bras Endocrinol Metabol 2014;58(5):411-33. doi: 10.1590/0004-2730000003388.

5. Hanley DA, Cranney A, Jones G, Whiting SJ, Leslie WD, Guidelines Committee of the Scientific Advisory Council of Osteoporosis C. Vitamin D in adult health and disease: a review and guideline statement from Osteoporosis Canada (summary). CMAJ Canadian Medical Association Journal 2010;182(12):1315-9. doi: https://dx.doi.org/10.1503/cmaj.091062.

6. Papaioannou A, Morin S, Cheung AM, Atkinson S, Brown JP, Feldman S, Hanley DA, Hodsman A, Jamal SA, Kaiser SM, et al. 2010 clinical practice guidelines for the diagnosis and management of osteoporosis in Canada: summary. Cmaj 2010;182(17):1864-73. doi: https://dx.doi.org/10.1503/cmaj.100771.

7. Khan A, Fortier M. Osteoporosis in menopause. J Obstet Gynaecol Can 2014;36(9):839-40. doi: 10.1016/s1701-2163(15)30489-8.

8. Pludowski P, Karczmarewicz E, Bayer M, Carter G, Chlebna-Sokol D, Czech-Kowalska J, Debski R, Decsi T, Dobrzanska A, Franek E, et al. Practical guidelines for the supplementation of vitamin D and the treatment of deficits in Central Europe - recommended vitamin D intakes in the general population and groups at risk of vitamin D deficiency. Endokrynol Pol 2013;64(4):319-27.

9. Rizzoli R, Branco J, Brandi ML, Boonen S, Bruyère O, Cacoub P, Cooper C, Diez-Perez A, Duder J, Fielding R, et al. Management of osteoporosis of the oldest old. Osteoporos Int 2014;25(11):2507-29. doi: 10.1007/s00198-014-2755-9.

10. Rizzoli R, Stevenson JC, Bauer JM, van Loon LJ, Walrand S, Kanis JA, Cooper C, Brandi ML, Diez-Perez A, Reginster JY, et al. The role of dietary protein and vitamin D in maintaining musculoskeletal health in postmenopausal women: a consensus statement from the European Society for Clinical and Economic Aspects of Osteoporosis and Osteoarthritis (ESCEO). Maturitas 2014;79(1):122-32. doi: https://dx.doi.org/10.1016/j.maturitas.2014.07.005.

11. Rizzoli R, Boonen S, Brandi ML, Bruyère O, Cooper C, Kanis JA, Kaufman JM, Ringe JD, Weryha G, Reginster JY. Vitamin D supplementation in elderly or postmenopausal women: a 2013 update of the 2008 recommendations from the European Society for

Clinical and Economic Aspects of Osteoporosis and Osteoarthritis (ESCEO). Curr Med Res Opin 2013;29(4):305-13. doi: 10.1185/03007995.2013.766162.

12. Lips P, Cashman KD, Lamberg-Allardt C, Bischoff-Ferrari HA, Obermayer-Pietsch B, Bianchi ML, Stepan J, Fuleihan GEH, Bouillon R. Current Vitamin D status in European and Middle East countries and strategies to prevent Vitamin D deficiency: A position statement of the European Calcified Tissue Society. European Journal of Endocrinology 2019;180(4):P23-P54. doi: http://dx.doi.org/10.1530/EJE-18-0736.

13. Makras P, Vaiopoulos G, Lyritis GP. 2011 guidelines for the diagnosis and treatment of osteoporosis in Greece. J Musculoskelet Neuronal Interact 2012;12(1):38-42.

14. Ip TP, Cheung SK, Cheung TC, Choi TC, Chow SL, Ho YY, Kan SY, Kung WC, Lee KK, Leung KL, et al. The Osteoporosis Society of Hong Kong (OSHK): 2013 OSHK guideline for clinical management of postmenopausal osteoporosis in Hong Kong. Hong Kong Med J 2013;19 Suppl 2:1-40.

15. Ibero American Concensus on Osteoporosis Sibomm. Osteoporosis: Prevention, Diagnosis, and Treatment; 2009.

16. Meeta, Digumarti L, Agarwal N, Vaze N, Shah R, Malik S. Clinical practice guidelines on menopause: An executive summary and recommendations. J Midlife Health 2013;4(2):77-106. doi: 10.4103/0976-7800.115290.

17. Dawson-Hughes B, Mithal A, Bonjour JP, Boonen S, Burckhardt P, Fuleihan GE, Josse RG, Lips P, Morales-Torres J, Yoshimura N. IOF position statement: vitamin D recommendations for older adults. Osteoporos Int 2010;21(7):1151-4. doi: https://dx.doi.org/10.1007/s00198-010-1285-3.

18. Kanis JA, Cooper C, Rizzoli R, Reginster JY, Scientific Advisory Board of the European Society for C, Economic Aspects of O, Osteoarthritis, the Committees of Scientific A, National Societies of the International Osteoporosis F. Executive summary of European guidance for the diagnosis and management of osteoporosis in postmenopausal women. Aging Clin Exp Res 2019;31(1):15-7. doi: https://dx.doi.org/10.1007/s40520-018-1109-4.

19. Rossini M, Adami S, Bertoldo F, Diacinti D, Gatti D, Giannini S, Giusti A, Malavolta N, Minisola S, Osella G, et al. Guidelines for the diagnosis, prevention and management of osteoporosis. Reumatismo 2016;68(1):1-39. doi: 10.4081/reumatismo.2016.870.

20. Orimo H, Nakamura T, Hosoi T, Iki M, Uenishi K, Endo N, Ohta H, Shiraki M, Sugimoto T, Suzuki T, et al. Japanese 2011 guidelines for prevention and treatment of osteoporosis--executive summary. Arch Osteoporos 2012;7(1-2):3-20. doi: 10.1007/s11657-012-0109-9.

21. The Jordan Osteoporosis Prevention Society**.** The Practical Guide for Management of Osteoporosis; 2016.

22. Yeap SS, Hew FL, Lee JK, Goh EML, Chee W, Mumtaz M, Damodaran P, Lim HH, Chan SP. The Malaysian Clinical Guidance on the Management of Postmenopausal Osteoporosis, 2012: A Summary. International Journal of Rheumatic Diseases 2013;16(1):30-40. doi: http://dx.doi.org/10.1111/1756-185x.12037.

23. Jassim NA, Adib G, Abdul Rahman YA, Gorial FI, Maghraoui A, Al Suhaili AR, Murtaji A, Otom A, Masri B, Saba E, et al. Pan Arab Osteoporosis Society Guidelines for Osteoporosis Management. Mediterr J Rheumatol 2017;28(1):27-32. doi: 10.31138/mjr.28.1.27.
24. Li-Yu J, Perez EC, Cañete A, Bonifacio L, Llamado LQ, Martinez R, Lanzon A, Sison M. Consensus statements on osteoporosis diagnosis, prevention, and management in the Philippines. Int J Rheum Dis 2011;14(3):223-38. doi: 10.1111/j.1756-185X.2011.01626.x.
25. Rusińska A, Płudowski P, Walczak M, Borszewska-Kornacka MK, Bossowski A, Chlebna-Sokół D, Czech-Kowalska J, Dobrzańska A, Franek E, Helwich E, et al. Vitamin D Supplementation Guidelines for General Population and Groups at Risk of Vitamin D Deficiency in Poland—Recommendations of the Polish Society of Pediatric Endocrinology and Diabetes and the Expert Panel With Participation of National Specialist Consultants and Representatives of Scientific Societies—2018 Update. Frontiers in Endocrinology 2018;9(246). doi: 10.3389/fendo.2018.00246.
26. Lorenc R, Gluszko P, Franek E, Jablonski M, Jaworski M, Kalinka-Warzocha E, Karczmarewicz E, Kostka T, Ksiezopolska-Orlowska K, Marcinowska-Suchowierska E, et al. Guidelines for the diagnosis and management of osteoporosis in Poland : Update 2017. Endokrynol Pol 2017;68(5):604-9. doi: https://dx.doi.org/10.5603/EP.2017.0062.
27. Raef H, Al-Bugami M, Balharith S, Moawad M, El-Shaker M, Hassan A, Al-Shaikh A, Al-Badawi I, King Faisal Specialist Hospital Osteoporosis Working G. Updated recommendations for the diagnosis and management of osteoporosis: a local perspective. Ann Saudi Med 2011;31(2):111-28. doi: https://dx.doi.org/10.4103/0256-4947.77502.

28. Bee CS, Chandran M. A Review of the 2008 Singapore Ministry of Health Clinical Practice Guidelines on Osteoporosis and an Update. Journal of the ASEAN Federation of Endocrine Societies 2014;27(2):159.

29. Lipschitz S, De Villiers T, Davey M. NOFSA statement on the appropriate management of osteoporosis in the elderly - Myth and reality. Journal of Endocrinology, Metabolism and Diabetes of South Africa 2016;21(3):S1-S2.

30. Varsavsky M, Rozas Moreno P, Becerra Fernandez A, Luque Fernandez I, Quesada Gomez JM, Avila Rubio V, Garcia Martin A, Cortes Berdonces M, Naf Cortes S, Romero Munoz M, et al. Recommended vitamin D levels in the general population. Endocrinol 2017;64 Suppl 1:7-14. doi: https://dx.doi.org/10.1016/j.endinu.2016.11.002.

31. The Taiwanese Osteoporosis Association**.** Taiwanese Guidelines for the Prevention and Treatment of Osteoporosis. In: Soong Y-KT, Keh-Sung; Yang, Rong-Sen;Wu, Chih-Hsing; Hwang, Jawl-Shan; Chan, Ding-Cheng, ed; 2011-2013:25.

32. Wu CH, McCloskey EV, Lee JK, Itabashi A, Prince R, Yu W, Li-Yu J, Chionh SB, Zhao Y, Shin CS, et al. Consensus of official position of IOF/ISCD FRAX initiatives in Asia-Pacific region. J Clin Densitom 2014;17(1):150-5. doi: 10.1016/j.jocd.2013.06.002.

33. Songpatanasilp T, Sritara C, Kittisomprayoonkul W, Chaiumnuay S, Nimitphong H, Charatcharoenwitthaya N, Pongchaiyakul C, Namwongphrom S, Kitumnuaypong T, Srikam W, et al. Thai Osteoporosis Foundation (TOPF) position statements on management of osteoporosis. Osteoporos Sarcopenia 2016;2(4):191-207. doi: 10.1016/j.afos.2016.10.002.

34. National Institute for Health and Care Excellence (NICE)**.** Vitamin D: supplement use in specific population groups; 2014.

35. National Institute for Health and Care Excellence (NICE)**.** Sunlight exposure: risks and benefits; 2016.

36. Compston J, Cooper A, Cooper C, Gittoes N, Gregson C, Harvey N, Hope S, Kanis JA, McCloskey EV, Poole KES, et al. UK clinical guideline for the prevention and treatment of osteoporosis. Archives of Osteoporosis 2017;12(1):43. doi: 10.1007/s11657-017-0324-5.

37. National Osteoporosis Guideline Group. NOGG 2017: Clinical guideline for the prevention and treatment of osteoporosis. UK; updated on 2018. pages: 36. https://www.sheffield.ac.uk/NOGG/NOGG%20Guideline%202017.pdf. Accessed on 05 June 2019.

38. Compston J, Bowring C, Cooper A, Cooper C, Davies C, Francis R, Kanis JA, Marsh D, McCloskey EV, Reid DM, et al. Diagnosis and management of osteoporosis in postmenopausal women and older men in the UK: National Osteoporosis Guideline Group (NOGG) update 2013. Maturitas 2013;75(4):392-6. doi: https://dx.doi.org/10.1016/j.maturitas.2013.05.013.

39. The US Preventive Services Task Force; Grossman DC, Curry SJ, Owens DK, Barry MJ, Caughey AB, Davidson KW, Doubeni CA, Epling JW, Jr., Kemper AR, et al. Vitamin D, Calcium, or Combined Supplementation for the Primary Prevention of Fractures in Community-Dwelling Adults: US Preventive Services Task Force Recommendation Statement. Jama 2018;319(15):1592-9. doi: https://dx.doi.org/10.1001/jama.2018.3185.

40. Watts NB, Adler RA, Bilezikian JP, Drake MT, Eastell R, Orwoll ES, Finkelstein JS, Endocrine S. Osteoporosis in men: an Endocrine Society clinical practice guideline. J Clin Endocrinol Metab 2012;97(6):1802-22. doi: https://dx.doi.org/10.1210/jc.2011-3045.

41. American Geriatrics Society Workgroup on Vitamin DSfOA. Recommendations abstracted from the American Geriatrics Society Consensus Statement on vitamin D for Prevention of Falls and Their Consequences. J Am Geriatr Soc 2014;62(1):147-52. doi: https://dx.doi.org/10.1111/jgs.12631.

42. The North American Menopause Society. Management of osteoporosis in postmenopausal women: 2010 position statement of The North American Menopause Society. Menopause. 2010;17(1):25-54.

43. Cosman F, de Beur SJ, LeBoff MS, Lewiecki EM, Tanner B, Randall S, Lindsay R, National Osteoporosis F. Clinician's Guide to Prevention and Treatment of Osteoporosis. Osteoporos Int 2014;25(10):2359-81. doi: https://dx.doi.org/10.1007/s00198-014-2794-2.

44. Camacho PM, Petak SM, Binkley N, Clarke BL, Harris ST, Hurley DL, et al. American Association of Clinicla Endocrinologists and American College of Endocrinology Clinical Practice Guidelines for the Diagnosis and Treatment of Postmenopausal Osteoporosis 2016. Endocr Pract. 2016;22(Suppl 4):1-42.

45. Sweet MG, Sweet JM, Jeremiah MP, Galazka SS. Diagnosis and treatment of osteoporosis. Am Fam Physician 2009;79(3):193-200.

46. Hauk L, American College of O, Gynecologists. ACOG releases practice bulletin on osteoporosis. American Family Physician. 2013;88(4):269-75.

47. Tuyen le D, Hien VT, Binh PT, Yamamoto S. Calcium and Vitamin D Deficiency in Vietnamese: Recommendations for an Intervention Strategy. J Nutr Sci Vitaminol (Tokyo) 2016;62(1):1-5. doi: https://dx.doi.org/10.3177/jnsv.62.1.

48. Wells A, Gray S. Following NICE guidance to take positive steps to prevent falls. Nursing & Residential Care 2013;15(11):729-32.

49. Elliott M. Taking control of osteoporosis to cut down on risk of fracture. Nursing Older People 2011;23(3):30-5.

50. Golden SH, Brown A, Cauley JA, Chin MH, Gary-Webb TL, Kim C, Sosa JA, Sumner AE, Anton B, Golden SH, et al. Health disparities in endocrine disorders: biological, clinical, and nonclinical factors--an Endocrine Society scientific statement. Journal of Clinical Endocrinology & Metabolism 2012;97(9):E1579-639. doi: 10.1210/jc.2012-2043.

51. Grasso D, Rafferty MA. Vitamin D: Implications of the Institute of Medicine Report for Clinical Practice. American Journal for Nurse Practitioners 2012;16(1/2):35-40.

52. Guertin E. Following MyPyramid the Plant-Based Way: Effects on Food Patterns and Nutrients. Nutrition Today 2012;47(1):7-22. doi: 10.1097/NT.0b013e3182435ae7.

53. Kopecky SL, Bauer DC, Gulati M, Nieves JW, Singer AJ, Toth PP, Underberg JA, Wallace TC, Weaver CM. Lack of Evidence Linking Calcium With or Without Vitamin D Supplementation to Cardiovascular Disease in Generally Healthy Adults: A Clinical Guideline From the National Osteoporosis Foundation and the American Society for Preventive Cardiology. Annals of Internal Medicine 2016;165(12):867-8. doi: https://dx.doi.org/10.7326/M16-1743.

54. Duque G, Lord SR, Mak J, Ganda K, Close JJT, Ebeling P, Papaioannou A, Inderjeeth CA. Treatment of Osteoporosis in Australian Residential Aged Care Facilities: Update on Consensus Recommendations for Fracture Prevention. Journal of the American Medical Directors Association 2016;17(9):852-9. doi: http://dx.doi.org/10.1016/j.jamda.2016.05.011.

55. Gielen E, Bergmann P, Bruyere O, Cavalier E, Delanaye P, Goemaere S, Kaufman JM, Locquet M, Reginster JY, Rozenberg S, et al. Osteoporosis in Frail Patients: A Consensus Paper of the Belgian Bone Club. Calcified Tissue International 2017;101(2):111-31. doi: http://dx.doi.org/10.1007/s00223-017-0266-3.

56. Gowan J, Roller L. Reducing falls risk and medication reviews. Australian Journal of Pharmacy 2009;90(1068):67-71.

57. Body JJ, Bergmann P, Boonen S, Devogelaer JP, Gielen E, Goemaere S, Kaufman JM, Rozenberg S, Reginster JY. Extraskeletal benefits and risks of calcium, vitamin D and anti-osteoporosis medications. Osteoporosis International 2012;23 Suppl 1:S1-23. doi: https://dx.doi.org/10.1007/s00198-011-1891-8.

58. Diab DL, Watts NB. Diagnosis and Treatment of Osteoporosis in Older Adults. Endocrinology and Metabolism Clinics of North America 2013;42(2):305-17. doi: http://dx.doi.org/10.1016/j.ecl.2013.02.007.

59. Kenny Lin. Interventions to prevent falls in community-dwelling older adults: Recommendation statement. American Family Physician 2018;98(4):252A-C.

60. Warren E. Prescribing for osteoporosis. Practice Nurse 2009;37(11):15-8.

61. U.S. Preventive Services Task Force. Screening for osteoporosis: U.S. Preventive Services Task Force recommendation statement. Annals of Internal Medicine 2011;154(5):356-65. doi: http://dx.doi.org/10.7326/0003-4819-154-5-201103010-00307.

62. Compston J, Cooper A, Cooper C, Francis R, Kanis JA, Marsh D, McCloskey EV, Reid DM, Selby P, Wilkins M. Guidelines for the diagnosis and management of osteoporosis in postmenopausal women and men from the age of 50 years in the UK. Maturitas 2009;62(2):105-8. doi: http://dx.doi.org/10.1016/j.maturitas.2008.11.022.

63. Essary AC, McNellis RJ. Three new recommendations from the US Preventive Services Task Force. Jaapa 2013;26(10):55-7. doi: https://dx.doi.org/10.1097/01.JAA.0000435002.24846.c6.

64. Gluszko P, Lorenc RS, Karczmarewicz E, Misiorowski W, Jaworski M, Working Group including the representatives of the Polish Associations of O, Traumatology RGRFMDLDA, Menopause ER, the Sg. Polish guidelines for the diagnosis and management of osteoporosis: a review of 2013 update. Pol Arch Med Wewn 2014;124(5):255-63.

65. Hanley DA, Cranney A, Jones G, Whiting SJ, Leslie WD, Cole DE, Atkinson SA, Josse RG, Feldman S, Kline GA, et al. Vitamin D in adult health and disease: a review and guideline statement from Osteoporosis Canada. Cmaj 2010;182(12):E610-8. doi: https://dx.doi.org/10.1503/cmaj.080663.

66. Moyer VA; The US Preventive Services Task Force. Vitamin D and calcium supplementation to prevent fractures in adults: U.S. Preventive Services Task Force recommendation statement. Annals of Internal Medicine 2013;158(9):691-6. doi: https://dx.doi.org/10.7326/0003-4819-158-9-201305070-00603.

67. Farrar MD, Webb AR, Kift R, Durkin MT, Allan D, Herbert A, Berry JL, Rhodes LE. Efficacy of a dose range of simulated sunlight exposures in raising vitamin D status in South Asian adults: implications for targeted guidance on sun exposure. Am J Clin Nutr 2013;97(6):1210-6. doi: https://dx.doi.org/10.3945/ajcn.112.052639.

68. McNellis R, Barnes KR. Vitamin d and calcium supplementation to prevent fractures in adults. American Family Physician 2014;89(11):897-8.

69. Gordon NP, Caan BJ, Asgari MM. Variation in Vitamin D supplementation among adults in a multi-race/ethnic health plan population, 2008. Nutrition Journal 2012;11 (1) (no pagination)(104). doi: http://dx.doi.org/10.1186/1475-2891-11-104.

70. Leidig-Bruckner G, Roth H, Bruckner T, Lorenz A, Raue F, Frank-Raue K. Are commonly recommended dosages for vitamin D supplementation too low? Vitamin D status and effects of supplementation on serum 25-hydroxyvitamin D levels-an observational study during clinical practice conditions. Osteoporosis International 2011;22(1):231-40. doi: 10.1007/s00198-010-1214-5.

71. Bryson DJ, Nichols JS, Ford AJ, Williams SC. The incidence of vitamin D deficiency amongst patients with a femoral neck fracture: are current bone protection guidelines sufficient ? Acta Orthopaedica Belgica 2013;79(4):470-3.

72. Sawka AM, Ismaila N, Raina P, Thabane L, Straus S, Adachi JD, Gafni A, Papaioannou A, Sawka AM, Ismaila N, et al. Hip fracture prevention strategies in long-term care: a survey of Canadian physicians' opinions. Canadian Family Physician 2010;56(11):e392-7.

73. Dawson-Hughes B, Looker AC, Tosteson ANA, Johansson H, Kanis JA, Melton ILJ. The potential impact of new National Osteoporosis Foundation guidance on treatment patterns. Osteoporosis International 2010;21(1):41-52. doi: http://dx.doi.org/10.1007/s00198-009-1034-7.

74. Ord H, Steele C. A baseline audit to evaluate the quality of nutritional provision delivered to fractured neck of femur inpatients. Journal of Human Nutrition & Dietetics 2011;24(4):398-9. doi: 10.1111/j.1365-277X.2011.01177_31.x.

75. Fung V, Kendler D. Preventing fractures in postmenopausal women: How to assess risk. Cmaj 2011;183(18):2129-31. doi: http://dx.doi.org/10.1503/cmaj.101054.

76. Hansen L, Tjonneland A, Koster B, Brot C, Andersen R, Lundqvist M, Christensen J, Olsen A. Sun exposure guidelines and serum vitamin D status in Denmark: The statusD study. Nutrients 2016;8 (5) (no pagination)(266). doi: http://dx.doi.org/10.3390/nu8050266.

77. Herrera Perez M, Rodriguez Rodriguez E, Alvisa Negrin JC, Pais Brito JL. Checklist for prevention of new hip fractures. Revista de Osteoporosis y Metabolismo Mineral 2013;5(1):7-14.

78. McLeod KM, Johnson S, Charturvedi R, Onge JS, Lionel A, Verma A. Bone mineral density screening and its accordance with Canadian clinical practice guidelines from 2000-2013: an unchanging landscape in Saskatchewan, Canada. Archives of Osteoporosis 2015;10 (1) (no pagination)(20). doi: http://dx.doi.org/10.1007/s11657-015-0227-2.

79. Tian M, Gong X, Rath S, Wei J, Yan LL, Lamb SE, Lindley RI, Sherrington C, Willett K, Norton R. Management of hip fractures in older people in Beijing: a retrospective audit and comparison with evidence-based guidelines and practice in the UK. Osteoporosis International 2016;27(2):677-81. doi: http://dx.doi.org/10.1007/s00198-015-3261-4.

80. O'Donnell S, Osteoporosis Surveillance Expert Working G. Screening, prevention and management of osteoporosis among Canadian adults. Health Promot Chronic Dis Prev Can 2018;38(12):445-54. doi: https://dx.doi.org/10.24095/hpcdp.38.12.02.

81. Rhodes LE, Webb AR, Fraser HI, Kift R, Durkin MT, Allan D, O'Brien SJ, Vail A, Berry JL. Recommended summer sunlight exposure levels can produce sufficient (> or =20 ng ml(-1)) but not the proposed optimal (> or =32 ng ml(-1)) 25(OH)D levels at UK latitudes. J Invest Dermatol 2010;130(5):1411-8. doi: https://dx.doi.org/10.1038/jid.2009.417.

82. Souberbielle JC, Body JJ, Lappe JM, Plebani M, Shoenfeld Y, Wang TJ, Bischoff-Ferrari HA, Cavalier E, Ebeling PR, Fardellone P, et al. Vitamin D and musculoskeletal health, cardiovascular disease, autoimmunity and cancer: Recommendations for clinical practice. Autoimmun Rev 2010;9(11):709-15. doi: https://dx.doi.org/10.1016/j.autrev.2010.06.009.

83. Papas E, Cluxton RJ. Vitamin D: beneficial for pain, fracture, and falls in long-term care residents? Annals of Long Term Care 2011;19(5):33-6.

84. Ströhle A, Hadji P, Hahn A. Calcium and bone health--goodbye, calcium supplements? Climacteric 2015;18(5):702-14. doi: 10.3109/13697137.2015.1016419.

85. Ben-Shoshan M. Vitamin D deficiency insufficiency and challenges in developing global vitamin D fortification and supplementation policy in adults. International Journal for Vitamin and Nutrition Research 2012;82(4):237-59. doi: http://dx.doi.org/10.1024/0300-9831/a000117.

86. Chakhtoura M, Rahme M, Chamoun N, El-Hajj Fuleihan G. Vitamin D in the Middle East and North Africa. Bone Reports 2018;8:135-46. doi: http://dx.doi.org/10.1016/j.bonr.2018.03.004.

87. Chen LR, Wen YT, Kuo CL, Chen KH. Calcium and vitamin D supplementation on bone health: Current evidence and recommendations. International Journal of Gerontology 2014;8(4):183-8. doi: http://dx.doi.org/10.1016/j.ijge.2014.06.001.

88. Nordin BEC, Lewis JR, Daly RM, Horowitz J, Metcalfe A, Lange K, Prince RL. The calcium scare-what would Austin Bradford Hill have thought? Osteoporosis International 2011;22(12):3073-7. doi: http://dx.doi.org/10.1007/s00198-011-1680-4.

89. Marcinowska-Suchowierska E, Walicka M, Talalaj M, Horst-Sikorska W, Ignaszak-Szczepaniak M, Sewerynek E. Vitamin D supplementation in adults - guidelines. Endokrynol Pol 2010;61(6):723-9.

90. Broy SB, Myers AK. Identifying and managing osteoporosis: an update. Journal of Musculoskeletal Medicine 2010;27(1):11-9.

91. Roush K. Prevention and Treatment of Osteoporosis in Postmenopausal Women: a review...Part two. AJN American Journal of Nursing 2011;111(8):26-37. doi: 10.1097/01.NAJ.0000403358.44058.f7.

92. Coughlan T, Dockery F. Osteoporosis and fracture risk in older people. Clinical Medicine 2014;14(2):187-91. doi: 10.7861/clinmedicine.14-2-187.

93. Bender D. Bisphosphonate and vitamin D supplementation for osteoporosis. Focus on Alternative & Complementary Therapies 2012;17(1):73-4. doi: 10.1111/j.2042-7166.2011.01138_16.x.

94. Schute LL. The diagnosis and treatment of primary osteoporosis according to current guidelines: impairments precede fractures...CME article about '... most common generalized disease of the skeleton... '. Deutsches Aerzteblatt International 2009;106(3):1-.

95. Aspray TJ, Francis RM. Here comes the sun: good news for bone health! Age & Ageing 2011;40(2):146-7. doi: ageing/afq164.

96. Weaver CM. Triennial Growth Symposium--Basis for establishment of 2011 vitamin D guidelines in humans. J Anim Sci 2014;92(3):893-8. doi: https://dx.doi.org/10.2527/jas.2013-7212.

97. Bonjour JP, Gueguen L, Palacios C, Shearer MJ, Weaver CM. Minerals and vitamins in bone health: the potential value of dietary enhancement. Br J Nutr 2009;101(11):1581-96. doi: https://dx.doi.org/10.1017/S0007114509311721.

98. Rosen CJ. Clinical practice. Vitamin D insufficiency. New England Journal of Medicine 2011;364(3):248-54. doi: https://dx.doi.org/10.1056/NEJMcp1009570.

99. Bouillon R, Carmeliet G. Vitamin D insufficiency: Definition, diagnosis and management. Baillieres Best Pract Res Clin Endocrinol Metab 2018;32(5):669-84. doi: https://dx.doi.org/10.1016/j.beem.2018.09.014.

100. Ebeling PR, Daly RM, Kerr DA, Kimlin MG. Building healthy bones throughout life: an evidence-informed strategy to prevent osteoporosis in Australia. Med J Aust 2013;199(7 Suppl):S1.

101. Boucher BJ. Vitamin D status and its management for achieving optimal health benefits in the elderly. Expert Review of Endocrinology and Metabolism 2018;13(6):279-93. doi: http://dx.doi.org/10.1080/17446651.2018.1533401.

102. Ruxton CHS, Derbyshire E. Health impacts of vitamin D: are we getting enough? Nutrition Bulletin 2009;34(2):185-97.

103. Aloia JF. Clinical Review: The 2011 report on dietary reference intake for vitamin D: where do we go from here? Journal of Clinical Endocrinology & Metabolism 2011;96(10):2987-96. doi: https://dx.doi.org/10.1210/jc.2011-0090.

104. Kuehn BM. USPSTF: Taking vitamin D and calcium doesn't prevent fractures in older women. Jama 2012;308(3):225-6. doi: https://dx.doi.org/10.1001/jama.2012.7955.

105. Vondracek SF. Managing osteoporosis in postmenopausal women. American Journal of Health-System Pharmacy 2010;67:S9-19. doi: 10.2146/ajhp100076.

106. Sareh P, Sourvine M, Steinle NI. Vitamin D and Calcium: Implications for Healthy Aging. Clinical Geriatrics 2011;19(12):29-34.

107. Carrier J. Clinical Q&A. Practice Nursing 2010;21(1):45-.

108. Gani LU, How CH. PILL Series. Vitamin D deficiency. Singapore Med J 2015;56(8):433-6; quiz 7. doi: https://dx.doi.org/10.11622/smedj.2015119.

109. Sheu A, Lopes LBA, Eisman J, Center JR. Osteoporosis management in 2017: still thin and fragmented. Internal Medicine Journal 2017;47(11):1329-30. doi: http://dx.doi.org/10.1111/imj.13598.

110. Manson JE, Bassuk SS. Calcium supplements: do they help or harm? Menopause (10723714) 2014;21(1):106-8. doi: 10.1097/GME.0b013e31829be090.

111. Lomangino K. The new calcium and vitamin D DRIs: a clinican's guide. Clinical Nutrition Insight 2011;37(2):1-5.

112. Chan SP, Chen JF, Chu LW, Van DP, Hosking D, Ip TP, Koh L, Kung A, Lai NS, Lau E, et al. Final Declaration of the Asian Metaforum on the Role of Vitamin D and the Management of Osteoporosis. Public Health Nutr 2009;12(4):578-80. doi: https://dx.doi.org/10.1017/S1368980008003224.

113. National Institue ofr Health and Care Excellence. Osteoporosis - quality standard. 2017. Available at: nice.org.uk/guidance/qs149; accessed on 5 August 2019.

114. National Institue of Health and Care Excellence. Hip fracture in adults - quality standard. 2012. Available at: nice.org.uk/guidance/qs16; accessed on 10 August 2019.

115. The Chinese Medical Association. The Chinese Osteoporosis Guidelines. Chinese general practice 2017.

116. Leon Schurman AB, Haraldo Clau S-Hermberg, Osvaldo D. Messina, Armando L. Negri, Ariel Sanchez, Claudio Gonzalez, Maria Diehl, Paula Rey, Julieta Gamba, Javier Chiarpenello, Maria Susana Moggia, Silvina Mastaglia Guidelines for the diagnosis, prevention and treatment of osteoporosis, 2012. MEDICINA (Buenos Aires) 2013(73):55-74.

117. Borissova A-M BM, Kolarov Z, Popivanov P, Shinkov A, Stoilov R, et al. Recommendations for the diagnosis, prevention and treatment of vitamin D insufficiency and deficiency. 2013.

S1. Search strategies

a. MEDLINE via Ovid

1. Vitamin D/
2. Cholecalciferol/
3. Ergocalciferols/
4. Vitamin D Deficiency/
5. Sunlight/
6. Ultraviolet Rays/
7. Vit* D.tw.
8. Ergocalciferol.tw.
9. Calciferol.tw.
10. Cholecalciferol.tw.
11. Sunlight.tw.
12. (Light adj3 expos*).tw.
13. UV.tw.
14. Ultraviolet.tw.
15. or/1-14
16. exp Calcium/
17. Calcium, Dietary/
18. calcium.mp.
19. or/16-18
20. 15 or 19
21. Diet/
22. "Diet, Food, and Nutrition"/
23. Diet, Vegetarian/
24. Diet, Vegan/
25. Diet, Western/
26. Diet Therapy/
27. Healthy Diet/
28. Food, Fortified/

29. Dietary supplements/
30. exp Dairy Products/
31. Shellfish/
32. Fishes/
33. exp Seafood/
34. Vegetables/
35. exp Vegetable Products/
36. Agaricales/
37. Diet*.tw.
38. (Diet* adj3 supplement*).tw.
39. (Fortified adj3 food*).tw.
40. Vegetable*.tw.
41. Mushroom*.tw.
42. Dairy.tw.
43. Milk.tw.
44. Cheese.tw.
45. Yog?urt.tw.
46. Seafood.tw.
47. Fish.tw.
48. Shellfish.tw.
49. or/21-48
50. 20 or 49
51. 20 and 49
52. exp clinical pathway/
53. exp clinical protocol/
54. exp consensus/
55. exp consensus development conference/
56. exp consensus development conferences as topic/
57. critical pathways/
58. exp guideline/

59. guidelines as topic/

60. exp practice guideline/

61. practice guidelines as topic/

62. health planning guidelines/

63. (guideline or practice guideline or consensus development conference or consensus development conference, NIH).pt.

64. (position statement* or policy statement* or practice parameter* or best practice*).ti,ab,kf,kw.

65. (standards or guideline or guidelines).ti,kf,kw.

66. ((practice or treatment* or clinical) adj guideline*).ab.

67. (C.P.G. or C.P.G.s).ti.

68. consensus*.ti,kf,kw.

69. consensus*.ab. /freq=2

70. ((critical or clinical or practice) adj2 (path or paths or pathway or pathways or protocol*)).ti,ab,kf,kw.

71. recommendat*.ti,kf,kw.

72. (care adj2 (standard or path or paths or pathway or pathways or map or maps or plan or plans)).ti,ab,kf,kw.

73. (algorithm* adj2 (screening or examination or test or tested or testing or assessment* or diagnosis or diagnoses or diagnosed or diagnosing)).ti,ab,kf,kw.

74. or/52-73

75. Osteoporosis/

76. Fractures, Bone/

77. Bone Density/

78. osteoporosis.tw.

79. fracture*,bone.tw.

80. bone density.tw.

81. bone health.mp.

82. 75 or 76 or 77 or 78 or 79 or 80 or 81

83. Adult/

84. Aged/

85. Middle Aged/

86. adult*.tw.

87. aged.tw.

88. middle aged.tw.

89. 83 or 84 or 85 or 86 or 87 or 88

90. 74 and 82 and 89

91. 50 and 90

b. Embase search strategy via Ovid

1. Vitamin D/
2. Cholecalciferol/
3. Ergocalciferols/
4. Vitamin D Deficiency/
5. Sunlight/
6. Ultraviolet Rays/
7. Vit* D.tw.
8. Ergocalciferol.tw.
9. Calciferol.tw.
10. Cholecalciferol.tw.
11. Sunlight.tw.
12. (Light adj3 expos*).tw.
13. UV.tw.
14. Ultraviolet.tw.
15. or/1-14
16. calcium/
17. calcium intake/
18. calcium.mp.
19. 16 or 17 or 18
20. 15 or 19
21. diet/
22. “Diet, Food, and Nutrition”/
23. Diet, Vegetarian/

24. Diet, Vegan/
25. Diet, Western/
26. Diet Therapy/
27. Healthy Diet/
28. Food, Fortified/
29. Dietary supplements/
30. exp Dairy Products/
31. Shellfish/
32. Fishes/
33. exp Seafood/
34. Vegetables/
35. exp Vegetable Products/
36. Agaricales/
37. Diet*.tw.
38. (Diet* adj3 supplement*).tw.
39. (Fortified adj3 food*).tw.
40. Vegetable*.tw.
41. Mushroom*.tw.
42. Dairy.tw.
43. Milk.tw.
44. Cheese.tw.
45. Yog?urt.tw.
46. Seafood.tw.
47. Fish.tw.
48. Shellfish.tw.
49. or/21-48
50. 20 or 49
51. exp clinical pathway/
52. exp clinical protocol/
53. exp consensus/
54. exp consensus development conference/
55. exp consensus development conferences as topic/

56. critical pathways/

57. practice guideline/

58. guidelines as topic/

59. health planning guidelines/

60. (position statement* or policy statement* or practice parameter* or best practice*).ti,ab,kw.

61. (standards or guideline or guidelines).ti,kw.

62. ((practice or treatment* or clinical) adj guideline*).ab.

63. (C.P.G. or C.P.G.s).ti.

64. consensus*.ti,kw.

65. consensus*.ab. /freq=2

66. ((critical or clinical or practice) adj2 (path or paths or pathway or pathways or protocol*)).ti,ab,kw.

67. recommendat*.ti,kw.

68. (care adj2 (standard or path or paths or pathway or pathways or map or maps or plan or plans)).ti,ab,kw.

69. or/51-68

70. Osteoporosis/

71. Fractures, Bone/

72. Bone Density/

73. osteoporosis.tw.

74. fracture*,bone.tw.

75. bone density.tw.

76. bone health.mp.

77. or/70-76

78. Adult/

79. Aged/

80. Middle Aged/

81. adult*.tw.

82. aged.tw.

83. middle aged.tw.

84. or/78-83

85. 69 and 77 and 84

86. 50 and 85

c. CINAHL

S1 TX "vitamin D" or "Cholecalciferol" or "Ergocalciferols" or "Vitamin D Deficiency"

S2 TX "sunlight" or "Ultraviolet Rays"or "Light adj3 expos*"

S3 (MH "Vit* D")

S4 (MH " Cholecalciferol")

S5 (MH "Sunlight+")

S6 S1 OR S2 OR S3 OR S4 OR S5

S7 TX "calcium" OR "calcium intake"

S8 (MH"calcium")

S9 S7 OR S8

S10 S6 OR S9

S11 TX "Diet" OR "Food" OR "Nutrition" OR "Diet, Vegetarian" OR "Diet, Vegan" OR "Diet, Western" OR "Diet therapy" OR "Healthy diet"

S12 TX "food, fortified" OR "dietary supplement*"

S13 TX "Dairy Product*" OR "Shellfish" OR "Fishes" OR "Seafood" OR "Vegetable*" OR "Vegetable Produc*" OR "Agaricales"

S14 (MH "diet*")

S15 (MH "Dietary Supplements+") OR (MH "Dietary Supplementation") OR (MH "Food, Fortified") OR (MH "Food, Formulated+")

S16 (MH "Vegetables+") OR (MH "Plants+")

S17 (MH "Mushroom, Edible")

S18 (MH "Dairy Products+") OR (MH "Milk Substitutes+") OR (MH "Yogurt") OR (MH "Vegetarianism") OR (MH "Lactobacillus Acidophilus") OR (MH "Cheese")

S19 (MH "Milk+") OR (MH "Soy Milk") OR (MH "Milk Substitutes+") OR (MH "Cultured Milk Products+")

S20 (MH "Cheese") OR (MH "Soy Foods+")

S21 (MH "Yogurt") OR (MH "Cultured Milk Products+")

S22 (MH "Seafood+")

S23 (MH "Fish")

S24 (MH "Shellfish")

S25 S11 OR S12 OR S13 OR S14 OR S15 OR S16 OR S17 OR S18 OR S19 OR S20 OR S21 OR S22 OR S23 OR S24

S26 S10 OR S25

S27 S10 AND S25

S28 TX "osteoporosis" or "bone health" or "bone density" or "fracture,bone"

S29 (MH "Osteoporosis+")

S30 (MH "Fractures+")

S31 S28 OR S29 OR S30

S32 TX "clinical pathway" OR "clinical protocol" OR "consensus" OR " critical pathways" OR "guideline" OR "practice guideline" OR "health planning guidelines"

S33 TX "position statement*" OR "policy statement*" OR "practice parameter*" OR "best practice"

S34 TX "standards" or "guideline" or "guidelines"

S35 TX "CPG" or "CPGs" or "clinical practice guideline*"

S36 TX " consensus*"

S37 TX "recommendat*

S38 TX "(care adj2 (standard or path or paths or pathway or pathways or map or maps or plan or plans))"

S39 (MH "Practice Guidelines")

S40 (MH "Guideline Adherence") OR (MH "Practice Guidelines") OR (MH "Dietary Reference Intakes")

S41 (MH "Consensus")

S42 (MH "Public Policy+") OR (MH "Health Policy Studies") OR (MH "Guideline Adherence")

S43 S32 OR S33 OR S34 OR S35 OR S36 OR S37 OR S38 OR S39 OR S40 OR S41 OR S42

S44 TX "adult*" or "aged" or "middle aged"

S45 (MH "Adult+")

S46 (MH "Aged+") OR (MH "Aged, 80 and Over+") OR (MH "Geriatric Nutritional Physiology")

S47 (MH "Middle Age")

S48 S44 OR S45 OR S46 OR S47

S49 S27 AND S31 AND S43 AND S48

S50 S26 AND S31 AND S43 AND S48

d. Other databases using key word search

National Guideline Clearinghouse – by Agency for Healthcare Research and Quality (AHRQ)

Vitamin D/calcium (in "Results with all of the words"); bone or osteoporosis (in "Results with exact phrases"); guideline, consensus, pathways, recommendations (in "Results with one of the phases")

Practice-based evidence in nutrition (PEN)

Searched vitamin D/calcium and osteoporosis or bone

National Institute for Health and Care Excellence (NICE)

Searched vitamin D or calcium separately

Guidelines International Network (GIN)

Searched each of the keywords one by one in the search bar: vitamin D, calcium, bone health, and osteoporosis
